# Supplementary material for: DrNote: An open medical annotation service
Source: PLOS Digit Health. 2022 Aug 15;1(8):e0000086. doi: 10.1371/journal.pdig.0000086 (PMC9931362; doi:10.1371/journal.pdig.0000086)
Supplement: Attachment — Submitted filename: RebuttalLetter.pdf [file pdig.0000086.s001.pdf]

Rebuttal Letter

2021-11-26

Dear editor, dear reviewers,

On behalf of all authors, we are grateful for your helpful in-depth feedback on our manuscript. We hope that you consider all raised points to be adequately addressed by our revised manuscript as described below and that the revised manuscript qualifies for publication.

Kind regards

Johann Frei

Editorial comments:

> 1. We ask that a manuscript source file is provided at Revision. Please upload your manuscript file as a .doc, .docx, .rtf or .tex. If you are providing a .tex file, please upload it under the item type ‘LaTeX Source File’ and leave your .pdf version as the item type ‘Manuscript’.

The .tex file of the manuscript will be uploaded.

> 2. We do not publish any copyright or trademark symbols that usually accompany proprietary names, eg (R), (C), or TM (e.g. next to drug or reagent names). Therefore please remove all instances of trademark/copyright symbols throughout the text, including "UMLS®" on page 12.

The manuscript has been updated accordingly.

> 3. Please update the completed 'Competing Interests' statement, including any COIs declared by your co-authors. If you have no competing interests to declare, please state "The authors have declared that no competing interests exist". Otherwise please declare all competing interests beginning with the statement "I have read the journal's policy and the authors of this manuscript have the following competing interests:"

The ‘Competing Interests‘ declaration will be uploaded.

Response to reviewers.

> Reviewer 1:
> Also in the concluding section, it would be great to get an assessment of performance of the tool vs purely data-driven approaches in English text as well as other non English text.
>
> Reviewer 2:
> + The primary weakness of this manuscript is that there are no empirical results which with to evaluate the performance of the proposed annotation framework. The manuscript focuses on describing the process by which the wiki dataset is created and various aspects of the general software platform (and how it uses/interfaces with OpenTapioca and Apache Solr) but there are no empirical evaluations or experiments to measure quality of the annotator.

This is a significant weakness and makes it difficult to evaluate the merits of the proposed contributions.
+ The domain shift from wiki text to EHR/clinical text is likely quite significant. A spot check with some MIMIC-III data (see below) reveals some of the limitations, but this needs to be characterized systematically using expert-labeled datasets. Restricting to medical entities in the WikiData KB is a valid use case, (for example, for tagging medical concepts in web data or consumer facing health literature this might be fine) but the cost of the domain shift needs to be measured
+ The multilinguality capability, while appealing, isn't motivated by results on multilingual datasets.

We addressed the core issue of the paper of lacking evaluation by adding annotation results for our method as well as for Apache cTAKES as reference method. Since our method is designed for multilingual and non-English settings, we focus on German data. Our clinical dataset is taken from another work on developing a German data-driven NER model (GERNERMED).
We don’t compare our method to this data-driven model: Although it was trained on the synthesized training set, it must be assumed that the test set is still biased by inherent dataset homogeneities. Furthermore, our GERNERMED model is currently under review.
Except for GERNERMED, no purely data-driven German model has been published.
We addressed the domain shift issue by further comparing our method with cTAKES on the Mantra GSC dataset which we were thankfully pointed to by reviewer 2.

Reviewer 2:
+ There are commercial solutions that handle Multilinguality (e.g., Amazon Comprehend Medical). It would be nice to include a baseline compared to such a service.

Although our primary focus lies on open methods that are freely accessible to academia, we investigated this point and signed up for Amazon Comprehend Medical on AWS. Although AWS does not mention it on the landing page for Comprehend Medical, apparently it does only support English texts according to the internal developer guide:
https://docs.aws.amazon.com/comprehend-medical/latest/dev/comprehendmedical-welcome.html

Reviewer 2:
+ The authors need to provide empirical measures of their systems performance by evaluating on some expert annotated (bio)medical datasets. Even an NER evaluation (vs. entity linking / NED, which is challenging here given the WikiData KG) would provide some sense of the annotator's term coverage and bound entity linking performance. There are a few parallel biomedical corpora that could be used:
- (biomedical) https://academic.oup.com/jamia/article/22/5/948/930067#210287674
- (biomedical) https://huggingface.co/datasets/scielo

Thank you for pointing this out. As mentioned above, we included the Matra GSC dataset in the context of domain shift evaluation.

Reviewer 2:
For clinical text, the situation is quite sparse (as noted by the authors) but there are clinical corpora in English that could be used to assess NER/term coverage and at least provide some empirical measurements of transitioning from wiki data to clinical/EHR text. Something like the 2018 n2c2 Adverse Drug Event (ADE) and Medication Extraction Challenge would work fine as an evaluation here.

Rebuttal Letter

For our recent EHR/clinical annotation results, we used the test set from the GERNERMED dataset, which essentially is a neural English to German translation of the n2c2 2018 ADE/Medication challenge dataset.

> Reviewer 1:
> The question I have is whether the approach used is novel compared to what already exists. It would be good to clarify this in the narrative.
> Reviewer 2:
> + There is considerable prior work on distant/weakly supervised, pseudo/silver-labeling and other methods for automatically generating training data that should be discussed as background and used to highlight the strengths of this work. These approaches are especially common in clinical concept recognition, but (as the authors note) this area is under-explored in multilingual settings. There are a few nice surveys of recent methods
> - "Modern Clinical Text Mining: A Guide and Review." Bethany Percha. Annual Review of Biomedical Data Science. 2021
> - "Clinical Concept Extraction: a Methodology Review." Fu et al. Journal of Biomedical Informatics. 2020
> - "Ontology-driven weak supervision for clinical entity classification in electronic health records." Fries et al. Nature Communications. 2021

Thank you for providing the reviews/surveys. We included the three publications in the related work section since they provide an excellent overview of various methodological concepts, especially for methods on sparse (labeled) data settings and dealing with data labeling “bootstrapping”/silver labeling.

> Reviewer 2:
> + (Line 280) The 1-2 weeks doesn't provide a very meaningful estimate of compute costs since in conflates multiple sources of compute/time costs (e.g., download time, data preprocessing, indexing, SVM training). Does the pipeline benefit from multiprocessing or support a distributed/cluster setup? It would be more helpful to describe the compute costs more precisely and provide more details, e.g., some definition of throughput based on number of candidate entities processed, # of wiki pages, etc

We added the table with our computation time measurements for various stages of the (pre-)processing pipeline, including indications on multicore support. We added the number of WikiData entities and Wikipedia pages to the manuscript text.

Tracking of modifications

Technical changes:

- We reduced the computation time for training by only extracting single sentences with relevant terms for the NIF file generation.
- We included the OpenTapioca selection features *MeSH descriptor ID* and *MeSH tree code* to cover a broader range of relevant WikiData terms. The OpenTapioca profile has been updated accordingly.
- We retrained the classifier with the updated OpenTapioca profile. (available at https://textmining.misit-augsburg.de)

Further manuscript changes:

- *MetaMaps* is mentioned as another relevant (English-focused) clinical text tool, similar to *cTAKES*.
- The extensive evaluation of the impact of selection features on the number of entities has been pruned in favor for the annoation performance evaluation section.
- Minor text changes that are implied by the technical changes described above.
