# Supplementary material for: DrNote: An open medical annotation service
Source: PLOS Digit Health. 2022 Aug 15;1(8):e0000086. doi: 10.1371/journal.pdig.0000086 (PMC9931362; doi:10.1371/journal.pdig.0000086)
Supplement: Attachment — Submitted filename: RebuttalLetter_V2.pdf [file pdig.0000086.s002.pdf]

Rebuttal Letter

2022-06-03

Dear editor, dear reviewers,

On behalf of all authors, we are grateful for your helpful in-depth feedback on our manuscript. We hope that you consider all raised points to be adequately addressed by our revised manuscript as described below and that the revised manuscript qualifies for publication.

Kind regards

Johann Frei

Editorial comments:

> 1. Please review your reference list to ensure that it is complete and correct. If you have cited papers that have been retracted, please include the rationale for doing so in the manuscript text, or remove these references and replace them with relevant current references. Any changes to the reference list should be mentioned in the rebuttal letter that accompanies your revised manuscript. If you need to cite a retracted article, indicate the article's retracted status in the References list and also include a citation and full reference for the retraction notice.

The reference list has been re-created in order to detect and avoid retracted papers, however non retracted paper was found. The citation style has been revised and URLs have been removed.

> Congratulation to the authors for substantially improving the manuscript in the first revision. As per the suggestion of the reviewer 1, I would strongly suggest the authors to add comparative analysis with the existing/traditional baseline.

As mentioned below, we tried to address this suggested point accordingly.

Response to reviewers.

> Reviewer 1:
> The authors are yet to bring out uniqueness of this approach compared to other available applications. Only providing references is not sufficient.

Unfortunately, the mentioned approaches such as TagMe from the related work section are difficult to compare to our solution in the context of medical use cases for the following reasons:
- TagMe and similar works like SWAT, WAT and REL are not based on WikiData entities and only use references to Wikipedia which is different from WikiData. This also includes the work on end-to-end neural entity linking [46].
We regard CHOLAN as the most relevant approach for entity linking to WikiData in this context. However, your attempts to reproduce the results described in the corresponding paper, and its GitHub repository in particular, in order to compare its performance scores failed since the authors did not publish the necessary model weights.
This issue is documented on their GitHub page, yet the authors did not answer the raised questions:
https://github.com/ManojPrabhakar/CHOLAN/issues/4
https://github.com/ManojPrabhakar/CHOLAN/issues/5

To avoid a comparison with only one related method, we ran and evaluated the test data on PubTator in addition to cTAKES. For demonstration purposes, we added five samples from the annotation results to better explain the differences of all approaches discussed in the paper.

Tracking of modifications

- Updated references
- Annotation results provided by PubTator added to the score table
- Annotation samples from the test set were added to highlight differences in annotation approaches
